# Subsequent Immunization of Pigs with African Swine Fever Virus (ASFV) Seroimmunotype IV Vaccine Strain FK-32/135 and by Recombinant Plasmid DNA Containing the CD2v Derived from MK-200 ASFV Seroimmunotype III Strain Does Not Protect from Challenge with ASFV Seroimmunotype III

**DOI:** 10.3390/vaccines11051007

**Published:** 2023-05-21

**Authors:** Alexey D. Sereda, Anna S. Kazakova, Sanzhi G. Namsrayn, Mikhail E. Vlasov, Irina P. Sindryakova, Denis V. Kolbasov

**Affiliations:** Federal Research Center for Virology and Microbiology (FRCVM), 601125 Volginsky, Petushki Area, Vladimir Region, Russia; namsrayn.szh@gmail.com (S.G.N.); vlasovmikhail1993@yandex.ru (M.E.V.); sindryakova.irina@yandex.ru (I.P.S.); kolbasovdenis@gmail.com (D.V.K.)

**Keywords:** African swine fever, seroimmunotype, CD2v, recombinant DNA

## Abstract

Understanding the immunological mechanisms of protection and the viral proteins involved in the induction of a protective immune response to the African swine fever virus (ASFV) is still limited. In the last years, the CD2v protein (gp110-140) of the ASFV has been proven to be a serotype-specific protein. Current work is devoted to the investigation of the possibility of creating protection against virulent ASFV strain Mozambique-78 (seroimmunotype III) in pigs previously vaccinated with vaccine strain FK-32/135 (seroimmunotype IV) and then immunized with the pUBB76A_CD2v plasmid, containing a chimeric nucleotide sequence from the CD2v protein gene (EP402R, nucleotides from 49 to 651) from the MK-200 strain (seroimmunotype III). Vaccination with the ASFV vaccine strain FK-32/135 protects pigs from the disease caused by the strain with homologous seroimmunotype—France-32 (seroimmunotype IV). Our attempt to create balanced protection against virulent strain Mozambique-78 (seroimmunotype III) by induction of both humoral factors of immunity (by vaccination with strain FK-32/135 of seroimmunotype IV) and serotype-specific cellular immunity (by immunization with the plasmid pUBB76A_CD2v of seroimmunotype III) was unsuccessful.

## 1. Introduction

African swine fever virus (ASFV) is responsible for causing acute hemorrhagic fever in both domestic pigs and wild boars, with a mortality rate of up to 100%. Developing a vaccine for ASFV has been challenging due to several unique properties. Firstly, the virus targets monocytes/macrophages, aggressively interfering with the host’s immune response regulation [1,2,3]. Secondly, the virus exhibits notable genomic plasticity, utilized in its vector transmission by the *Ornithodoros* soft ticks. Lastly, the virus displays a considerable phenotypic variation, including virulence, serotypic and immunotypic characteristics, and hemadsorption-inducing ability [4,5,6,7,8,9,10,11,12,13,14]. 

Recently, ASFV strains have been classified into nine groups based on their seroimmunotype (from I to IX). *In vitro*, the grouping was based on a comprehensive assessment of ASFV isolates/strains by antigenic properties in haemadsorption inhibition assay using type-specific sera. In contrast, *in vivo*, this grouping used the data of an immunological test (bioassay) on pigs inoculated with attenuated strains [7,8,9,10,11].

The research on developing protection against ASF has led to the discovery of attenuated strains of the first eight seroimmunotypes. However, these attenuated strains exhibited differences in several biological characteristics, including the duration and level of viremia and the timing of virus-specific protection formation. For example, an attenuated FK-32/135 strain (seroimmnotype IV) at a dose of 10^4.0^ HAU_50_ created protection on the 7th–10th days in 92–100% of pigs, while the strain MK-200 (seroimmunotype III) at the same dose protected 50% of animals on days from 7 to 10, and only on 14th day it created protection in 82–92% of pigs [11].

A protective immune response develops in the natural hosts and domestic pigs that recover from the infection and are challenged with a homologous isolate [15]. However, the mechanisms of this protective response are not entirely understood. Several reports have demonstrated the important role of antibodies in protective immunity through passive antibody transfer experiments [16,17]. In addition, immunization with the viral structural proteins p32 and p54 was found to be insufficient for protection, with 50% of immunized animals surviving a virulent virus challenge, although later developing clinical signs [18]. These results indicate that complete protection also requires a cellular component of the immune response. Indeed, ASFV-specific cytotoxic T lymphocytes (CTL) that can lyse infected macrophages were identified after infection with less virulent isolates [19]. Also, the involvement of CD8^+^ T cells in protection was demonstrated recently [20]. In addition, increased natural killer cell activity was also correlated with protection following inoculation with the ASFV/NH/P68 isolate. Finally, multiple serological and cellular immune response targets have been demonstrated [21,22]. Such complexity of the protective immune response makes the development of an effective vaccine a challenging task, and thus it is necessary to expand current knowledge about ASFV proteins.

The CD2v ASFV gene or EP402R (also known as 8DR) encodes for a structural transmembrane glycoprotein that has been shown to mediate haemadsorption [23,24]. Previous studies have obtained information about the role of CD2v protein in virulence and protection using different virus models [25,26]. Interestingly, some ASFVs have truncated or interrupted CD2v (EP402R) and, as a result, have not demonstrated haemadsorbing ability. Some non-haemadsorbing ASFV strains have attenuated phenotype and have been used as a model for vaccine research [27]. Of all known proteins of the virus, the property of serotype specificity has been proven only for the gp110-140 glycoprotein (CD2v) [8]. Immunization with recombinant baculovirus carrying the CD2v gene of the ASFV protected pigs from subsequent challenges with a virulent strain [28]. Presumably, this protein plays a significant role in the activation of CTLs.

A possible solution to the problem of the complexity of the protective immune response to the ASFV virus might be recombinant DNA constructs that include genes of viral proteins involved in the induction of both humoral and cellular mechanisms of specific protection [29,30,31]. Immunization of pigs with a recombinant plasmid containing merged p30 and p54 genes of structural proteins did not induce any immune response that is detectable by ELISA—including a gene fragment encoding the extracellular domain of a glycoprotein sCD2v into the plasmid resulted in a significant immune response in the vaccinated pigs. However, the DNA vaccine could not confer protection against lethal viral challenges [32]. DNA vaccines encoding p30 and p54 fused (pCMV-PQ) induced good antibody responses in mice but not in pigs, where they were undetectable. DNA immunization in pigs could be exponentially improved by adding the extracellular domain of HA (sHA) to the vaccine-encoded antigens. Pigs immunized with pCMVsHAPQ-induced strong humoral and cellular responses, even though no protection was afforded against the lethal ASFV challenge. And second that 33% of the pigs immunized with pCMV-UbsHAPQ, encoding the same three ASFV antigens fused to ubiquitin, survived the lethal challenge. Protection was afforded in the absence of vaccine-induced antibodies. More importantly, it correlated with the proliferation of antigen-specific CD8^+^ T-cells recognizing two previously undescribed 9-mer epitopes, mapping within the sHA [33]. The importance of ubiquitination was confirmed by the results of the immunization of pigs with ASFV^Ublib^ (a DNA library containing short coding fragments from the ASFV genome connected to a ubiquitin gene). After infection with the homologous ASFV strain, partial protection of animals was reached in the absence of detectable antibodies, confirming the hypothesis of the crucial role of CD8^+^-T cells in protection against ASFV [34].

Earlier, we reported on a design of the recombinant plasmids with fragments of genes encoding viral proteins of ASFV involved in the induction of protective immune response, namely p30, p54, and CD2v from the attenuated strain MK-200 (seroimmunotype III). Immunization of pigs with autologous leukocytes transfected with these recombinant plasmids did not show any detectable antibody production and failed to protect against challenge by the homologous virulent virus [35]. Triple immunization of pigs with a mix of recombinant plasmids, each of which contained a ubiquitin gene and parts of genes encoding ectodomains of proteins p30, p54, and CD2v from the MK-200 strain, led to the formation of the virus-specific CTLs, but not to the formation of detectable virus-specific antibodies. After the intramuscular infection with the virulent strain Mozambique-78, all immunized pigs died within 4 to 7 days [36]. These results demonstrated that looking for experimental approaches to induce both humoral and cellular protection mechanisms is necessary.

This paper is devoted to the investigation of the possibility of creating protection against the ASFV strain Mozambique-78 (seroimmunotype III) in pigs previously vaccinated with the strain FK-32/135 (seroimmunotype IV) and then immunized with the pUBB76A_CD2v plasmid, containing a chimeric nucleotide sequence from the CD2v protein gene (EP402R, nucleotides 49-651) from the MK-200 strain (seroimmunotype III).

## 2. Materials and Methods

### 2.1. Viruses and Cells

The ASFV strains were obtained from the Federal Research Center for Virology and Microbiology (FRCVM) collection of microorganisms: the virulent strain Mozambique-78 (M-78) and the vaccine strain MK-200 derived from it (seroimmunotype III, genotype V), and the virulent strain France-32 (F-32) and the vaccine strain FK-32/135 derived from it (seroimmunotype IV, genotype I) [7].

The infectivity of the ASFV was determined by titration in primary blood leukocytes of swine (PBLS) and pig bone marrow cells (PBMC) cultures, as previously described [37]. The virus titrations were determined by haemadsorption and were calculated by the method of B.A. Kerber was modified by I.P. Ashmarin and expressed as 50% haemadsorbing units (HAU_50_) per ml (HAU_50_/mL) [38].

### 2.2. Animal Experiments and Ethics Statement

Both female and male pigs of a large white breed 3–4 months old weighing 35–40 kg from the Experimental Animal Preparation Sector of the FRCVM were used. Experiments involving animals and viruses were performed in accordance with the National Institutes of Health’s Guide for the Care and Use of Laboratory Animals. They were approved by the institutional animal care and use and institutional biosafety committees at FRCVM. The pigs were kept and euthanized in accordance with the protocol Guide for the Care and Use of Laboratory Animals, AVMA Guidelines [39], and all efforts were made to minimize suffering. The scheme of the experiment is provided in Table 1.

Pigs No. 1, 2, and No. 5–8 were vaccinated intramuscularly with 1.0 mL of ASFV strain FK-32/135 at a dose of 10^4.0^ HAU_50_ (day 0). On days 3 and 17, pigs No. 5–7 and No. 10–12 were immunized with the recombinant plasmid pUBB76A_CD2v containing a chimeric nucleotide sequence from the CD2v protein gene (EP402R, nucleotides 49–651) from strain MK-200 and monoubiquitin B (nucleotides 1–226) [36]. One immunizing dose for each pig contained 1.0 mg of plasmid DNA in a final volume of 1.5 mL of phosphate-buffered saline (PBS) at pH 7.2. The total volume was divided into three equal parts. It was administered in 0.5 mL doses intramuscularly into a trapezoid muscle of the neck, into the quadriceps femoris muscle of a hip, and subcutaneously into an ear. On day 24, pigs No. 1–4 and No. 12 were infected intramuscularly with ASFV strain France-32 at a dose of 10^3.0^ HAU_50_ and pigs No. 5–11—with ASFV strain Mozambique-78 at a dose of 10^3.0^ HAU_50_.

During the experiment, blood samples were collected from the cranial *vena cava* of pigs on days 0, 10, 17, and 24. These samples were divided into two groups: 5 mL of blood samples were taken into test tubes with a coagulant for receiving serums. In contrast, 3–5 mL of blood was taken in test tubes with anticoagulant lithium heparin for determination of viremia levels and counting of the number of cells secreting γ-interferon (IFNγ) on days 27, 29, 31, 34, 36, 38, 40, and 42, 5 mL of blood samples were collected from each live animals to obtain sera for the determination of virus-specific antibodies. Additionally, 3 mL of blood samples were taken in test tubes with the anticoagulant lithium-heparin to measure the viremia levels. The body temperature of the pigs was measured rectally daily.

### 2.3. Detection of Virus-Specific Antibodies

ASFV-specific antibodies in pig serum were detected using the OIE-recommended ELISA protocol [37]. ELISA antigens were prepared from uninfected and infected cells grown in pig serum [40]. PBMC were cultured in 75 mL vials at 37 °C in a modified Dulbecco Eagle medium (DMEM) with the addition of 2 mM L-glutamine, 100 U/mL gentamicin, essential amino acids, and 5% thermo-inactivated pig serum. After 48 h, the unattached cells were drained, adherent cells were rinsed with buffered saline solution, infected with ASFV strain MK-200 with a multiplicity of 0.1 HAU_50_/cell, and then covered with the initial volume of the culture medium. After 48 h, ASFV-infected and uninfected adherent cell cultures were separately harvested, at different times, washed with 0.34 M sucrose in 5 mM Tris–HCl (pH 8.0), resuspended in 0.067 M sucrose and held at 0 °C for 10 min to allow the cells to swell. Cells were lysed by adding the detergent Nonidet P40 at a final concentration of 1% (*w*/*v*) and held for 10 min at 0 °C. A 1/7 volume of 64% (*w*/*v*) sucrose to 0.4% Tris–HCl (pH 8.0) was added, and nuclei were pelleted at 1000 g for 10 min at 4 °C. The supernatants (cytoplasmic fraction) were treated with 2 mM ethylene-diamine–tetra acetic acid (EDTA), 0.05 M 2-mercaptoethanol and 0.5 M NaCl. After 15 min at 25 °C, the mixtures were centrifuged at 100,000 g for 1 h at 4 °C over a 20% (*w*/*w*) sucrose cushion in 50 mM Tris–HCl (pH 8.0). An indirect ELISA test used the fractions eluted from the sucrose layers as soluble ASFV-specific (positive control) and normal (negative control) antigens.

MaxiSorp™ ELISA plates (Nunc™, Thermo Fisher Scientific Inc., Waltham, MA, USA) were coated with either infected cell or uninfected cell antigen (50 μL per well) diluted (1–10 μg/mL) in coating buffer (50 mM sodium carbonate/bicarbonate buffer, pH 9.6) and incubated overnight at 4 °C. The wells were washed three times with PBS plus 0.05% Tween 20 and blocked with PBS plus 10% milk (200 μL per well) at 37 °C for 1 h. After blocking, the plates were washed five times as described above and incubated for 1 h at 37 °C with pig sera diluted 1:20 in PBS plus 5% milk (50 μL per well). The wells of plates were washed five times, as mentioned above. Antibody-positive and negative controls were duplicated on each ELISA plate. Plates were incubated at 25 °C for 1 h and then washed five times. The presence of positive sera was detected using a peroxidase-conjugated anti-pig IgG at a 1/10,000 dilution (Sigma-Aldrich, St. Louis, MO, USA) as a secondary antibody and soluble 3,3,5,5-tetramethylbenzidine (TMB) as a specific peroxidase substrate (Sigma-Aldrich). Reactions were stopped with 1 N H_2_SO_4_ (Sigma-Aldrich), and the ELISA plates were read at a wavelength of 450 nm. The results were represented as the average absorbance (optical density [OD] values) of duplicates [41].

### 2.4. IFNγ ELISpot

Quantities of IFNγ-secreting T-cells were determined using enzyme-linked immune absorbent spot (ELISpot). The analysis was carried out over three repetitions. First, whole blood samples were collected into Vacutainer test tubes containing lithium heparin (BD Biosciences, Fisher Scientific, Pittsburgh, PA, USA). Next, mononuclear cells were isolated from peripheral blood by gradient centrifugation with the use of Histopaque-1077 (Sigma-Aldrich), washed twice, and resuspended in a serum-free CTL-test culture medium (Cellular Technology Limited, Cleveland, Ohio, USA) containing 2 mM of L-glutamine and 80 mg/L of gentamycin. The final cell density was 5 × 10^5^ cells/mL. Detection of ASFV-induced production of IFNγ in the culture of peripheral blood mononuclear cells (PBMC) was carried out according to the instructions of the commercial kit Pig IFNγ Single-Color ELISPOT (ImmunoSpot^®^, Clevelend, OH, USA) [42]. Briefly, monoclonal anti-IFNγ antibodies with a concentration of 5 µg/mL in 100 µL of PBS were absorbed in wells overnight at 4 °C. Next, strips were washed with PBS, and 5 × 10^5^ PBMC were added to the wells. Finally, the ASFV strains France-32 or Mozambique-78 were added to wells at a dose of 10^5.0^ HAU_50_. Background secretion of IFNγ by mononuclear cells in the presence of the culture medium acted as a negative control. After 24 h of cultivation at 37 °C (in the presence of 5% CO_2_), cells were removed, and a biotinylated secondary anti-pork IFNγ antibody was added to the wells, followed by incubation for 2 h at room temperature. Then, strips were washed and incubated at room temperature with streptavidin–peroxidase (30 min) and insoluble 3,3′,5,5′-tetramethylbenzidine (TMB) (15 min). The reaction was stopped by carefully washing the strips with the distilled water. To calculate the number of cells secreting IFNγ, the spots in a non-stimulated well were calculated, and their number was subtracted from the number of spots in wells stimulated with the virus. Results were expressed as the quantity of the reacting cells in 10^6^ PBMC.

## 3. Results

**Figure 1:**
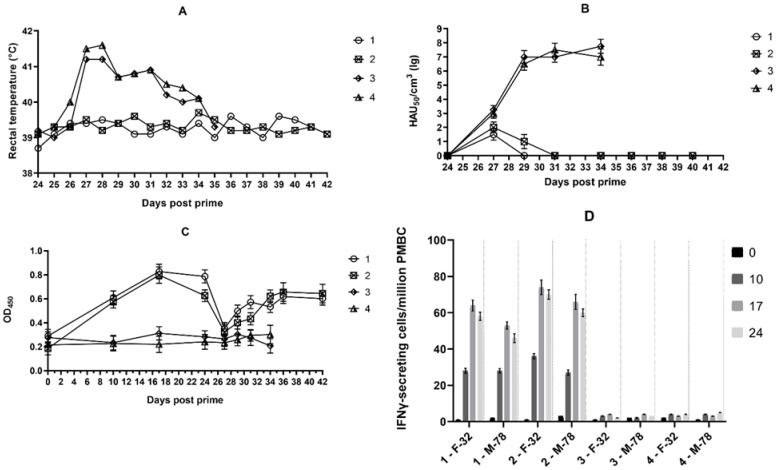
Dynamics of body temperature, viremia, virus-specific antibody response and IFNγ production in animals in the first group. Pigs No. 1 and No. 2 were vaccinated with the ASFV strain FK-32/135 (seroimmunotype IV) on day 0, and No. 3 and No. 4 were not vaccinated. On day 24 post-immunization, all No. 1–4 animals were infected with the virulent ASFV strain France-32 (seroimmunotype IV): (**A**) rectal body temperature of animals after infection; (**B**) post-infection viremia; (**C**) ASFV-specific antibodies in serum (dilution 1:20) in indirect ELISA (antibody levels are represented by mean optical density); and (**D**) a number of ASFV-induced IFNγ secreting cells per million of PBMC obtained from pigs on days 0, 10, 17, and 24. ASFV-induced IFNγ secreting cells were stimulated by the virulent strains France-32 (F-32) (seroimmunotype IV) and Mozambique-78 (M-78) (seroimmunotype III) and measured using ELISpot.

The dynamics of body temperature and viremia from group 1 animals are shown in Figure 1A and Figure 1B, respectively. Pigs No. 1 and 2, vaccinated with FK-32/135, had a body temperature within the norm for 18 days after infection with strain France-32, and clinical manifestations of the disease were absent. On day 27 post-immunization (i.e., on the third day after infection by virulent strain), insignificant viremia was noted (10^1.50^–10^2.00^ HAU_50_/mL). From day 31 to day 42, ASFV was not detected in the pig’s blood. Pigs No. 3 and 4, not vaccinated with FK-32/135, manifested an acute ASFV infection on days 27 to 36 (fever, fatigue, and anorexia). Viremia was 10^3.00^ HAU_50_/mL on day 27 and gradually rose to 10^6.75^–10^7.75^ HAU_50_/mL from day 29 to day 36. The animals were subjected to euthanasia in an agonal state.

ASFV-specific antibodies in pigs No. 1 and No. 2 (vaccinated with strain FK-32/135) were detectable from day ten to 24 (Figure 1C). On days 27–31 post-immunization and three-seven days post-infection with virulent strain France-32, pigs No. 1 and No. 2 decreased the level of virus-specific antibodies, followed by a slight increase on day 34 with an exit to a plateau. No virus-specific antibodies were found in unvaccinated pigs No. 3 and No. 4, including the period after infection with France-32.

Specific IFNγ-secreting T-cells were found in pigs No. 1 and No. 2 when stimulated by the ASFV strains France-32 or Mozambique-78. Both animals showed increased T-cellular response on days 10–24 following the vaccination (Figure 1D). The maximum values were recorded on day 17. No specific IFNy-secreting T-cells were found in unvaccinated animals No. 3 and 4.

**Figure 2:**
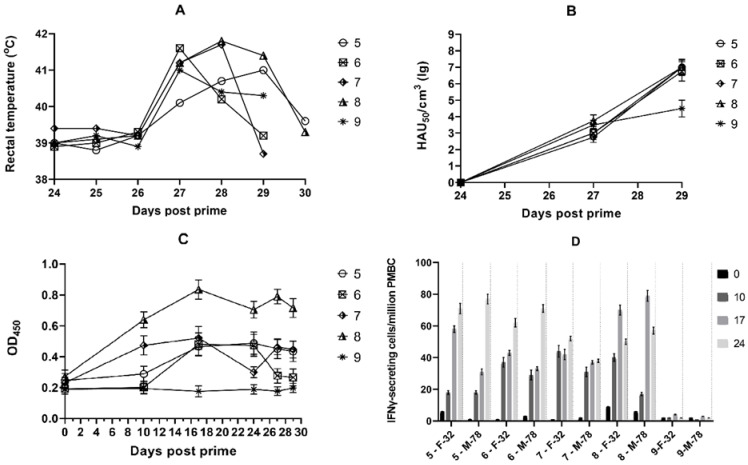
Dynamics of body temperature, viremia, virus-specific antibody response and IFNγ production in animals from the second group. Pigs No. 5–8 were vaccinated with the ASFV strain FK-32/135 (seroimmunotype IV) on day 0, and pigs No. 5–7 were immunized twice (on days 3 and 17) with the recombinant plasmid pUBB76A_CD2v (encoding the CD2v gene from a strain of MK-200 derived from seroimmunotype III). Pig No. 9 was not vaccinated. On day 24 post-immunization, all animals No. 5–9 were infected with the virulent ASFV strain Mozambique-78 (M-78) (seroimmunotype III): (**A**) rectal body temperature of animals after infection; (**B**) post-infection viremia; (**C**) ASFV-specific antibodies in serum (dilution 1:20) in indirect ELISA; (**D**) number of ASFV-induced IFNγ-secreting cells per million of PBMC obtained from pigs on days 0, 10, 17, and 24. ASFV-induced IFNγ-secreting cells were stimulated by the virulent strain France-32 (F-32) (seroimmunotype IV) and Mozambique-78 (M-78) (seroimmunotype III) and measured using ELISpot.

The dynamics of body temperature and viremia in pigs of group 2 (pigs No. 5–9) infected on day 24 with the virulent ASFV strain Mozambique-78 are presented in Figure 2A and Figure 2B, respectively.

From day 27 post-immunization (on the third day after infection with virulent strain Mozambique-78), all pigs of group 2 demonstrated an acute form of ASFV infection with temperatures of 41.0–41.8 °C, and on days 29 to 30, the animals were in agonal state and were euthanized. On day 27, viremia was in the range of 10^2.75^–10^4.00^ HAU_50_/mL. On days 29 and 30, pigs displayed viremia in the 10^6.75^–10^7.75^ HAU_50_/mL range. Pig No. 8, vaccinated with the strain FK-32/135, same as pigs No. 3 and No. 4 from group 1, showed a higher level of antibodies than pigs No. 5–7, which were vaccinated with strain FK-32/135 and then immunized twice with the pUBB76A_CD2v plasmid (encoding the CD2v from a strain of MK-200) (Figure 2C). IFNγ production increased in animals of the second group No. 5–8, indicating the induction of the T-cellular response after vaccination with strain FK-32/135 and during immunization with the pUBB76A_CD2v plasmid (Figure 2D). Animals No. 5–7, immunized with the pUBB76A_CD2v plasmid after vaccination, had the maximum production of IFNy not on day 17 but on day 24.

**Figure 3:**
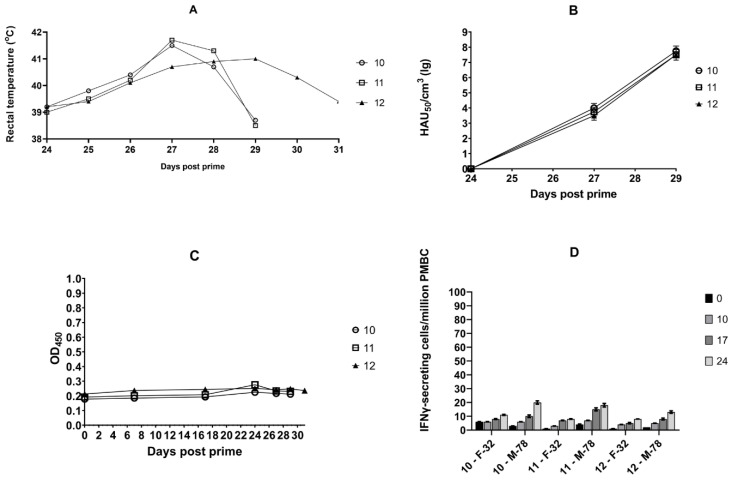
Dynamics of body temperature, viremia, virus-specific antibody response and IFNγ production in animals from the third group. Pigs No. 10–12 were immunized twice (on days 3 and 17) with the recombinant plasmid pUBB76A_CD2v (encoding the CD2v gene from a strain of MK-200 derived from seroimmunotype III). On day 24 after the start of the experiment, animals No. 10 and No. 11 were infected with the virulent ASFV strain Mozambique-78 (M-78) (seroimmunotype III), No. 12—with the virulent ASFV strain France-32 (seroimmunotype IV): (**A**) rectal body temperature of animals after infection; (**B**) post-infection viremia; (**C**) ASFV-specific antibodies in serum (dilution 1:20) in indirect ELISA; (**D**) a number of ASFV-induced IFNγ-secreting cells per million of PBMC obtained from pigs on days 0, 10, 17, and 24. ASFV-induced IFNγ-secreting cells were stimulated by the virulent strain France-32 (F-32) (seroimmunotype IV) and Mozambique-78 (M-78) (seroimmunotype III) and measured using ELISpot.

Group 3 showed similar body temperature and viremia dynamics to animals from Group 2 and intact animals from Group 1 (Figure 3A,B).

**Figure 4:**
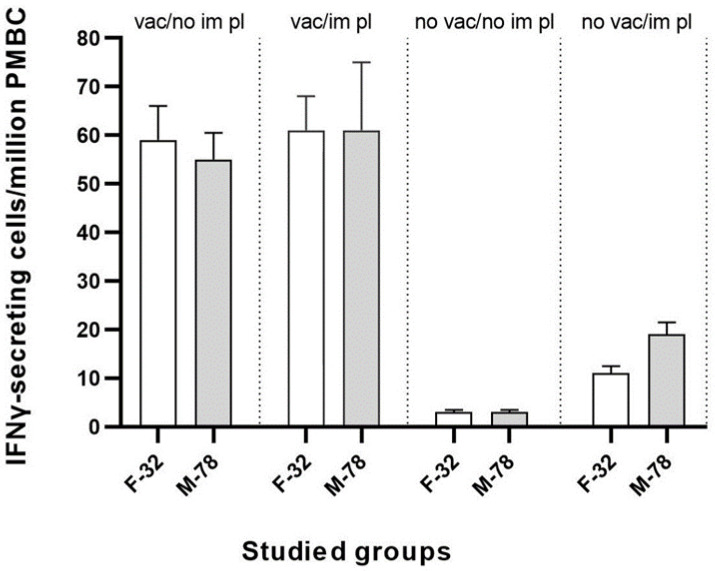
Detection of ASFV-specificT-cells on day 24 after the start of the experiment by an IFNγ-ELISPOT in pigs vaccinated (vac)/non-vaccinated (no vac) with the ASFV strain FK-32/135 (seroimmunotype IV) and immunized with the recombinant plasmid pUBB76A_CD2v (encoding the CD2v gene from a strain of MK-200 derived from seroimmunotype III) (im pl)/non-immunized (no im pl). ASFV-induced IFNγ-secreting cells were stimulated by the virulent strain France-32 and Mozambique-78.

There was no significant increase in the level of virus-specific antibodies in pigs immunized with the recombinant plasmid pUBB76A_CD2v (Figure 3C). However, animals in group 3 showed an important (*p* < 0.05) increase in IFNγ production by day 24 after the start of the experiment, which can be correlated with the induction of a T-cell response after immunization with the pUBB76A_CD2v plasmid (Figure 3D). In addition, the induction of T-cell response to the homologous strain Mozambique-78 was significantly higher than to the strain France-32, at a significance level of *p* < 0.05 (Figure 4).

There were no significant differences between the studied groups of pigs vaccinated with the ASFV strain FK-32/135 and immunized/non-immunized with the recombinant plasmid pUBB76A_CD2v.

## 4. Discussion

Among the selected conditional vaccine strains against the I-V seroimmunotypes of the ASFV, the strain FK-32/135 was the most effective in protection against isolates of seroimmunotype IV [11]. Within three days after its administration, virus-specific antibodies were detected in the blood of pigs by radioimmunoprecipitation; after six days, CTLs were detected, and animals became resistant to infection with virulent strains and isolates of the seroimmunotype IV, including strain France-32 [22].

As expected, the immunization of pigs with ASFV strain FK-32/135 conferred protection against infection with the homologous strain France-32. After the infection with France-32, the ASF virus was detectable in the blood of these animals starting from days 3 to 5. In contrast, unvaccinated pigs (No. 3 and No. 4) succumbed to the virulent France-32 strain within 10–11 days post-infection. Our findings align with previous reports on the efficacy of OUR T88/3 and NH/P68 isolates in protecting vaccinated animals in group 1 [27,43]. Strain FK-32/135 exhibits unique characteristics, as it seldom induces chronic ASF *in vivo* and elicits «loose» hemadsorption *in vitro*. The dynamics of ASFV-specific antibodies were consistent with previous studies, with detectable levels from 7 to 12 days post-infection [42,43,44].

Additionally, vaccinated pigs exhibited higher levels of IFNγ secretion on days 17 and 24 compared to day 10. The production of IFNγ by PBMC infected with the seroimmunotype homologous strain France-32 was slightly higher (*p* < 0.05) than by PBMC infected with the heterologous seroimmunotype strain Mozambique-78. Finally, the infection of pigs from group 2 with the ASFV strain Mozambique-78 resulted in an acute form of the disease with high temperatures and death within 5–6 days. In animals vaccinated with strain FK-32/135, No. 8 from group 2 and No. 1 and No. 2 from group 1, the antibody-level readings were higher than in animals No. 5–7 from group 2, which were immunized twice with the pUBB76A_CD2v plasmid (encoding the CD2v from a strain of MK-200) after vaccination with strain FK-32/135. Another finding is the shift of the maximum IFNγ production from day 17 to day 24 in the group of pigs that underwent pUBB76A-CD2v immunization twice after vaccination. Such differences indicate that pUBB76A-CD2v immunization causes clear and measurable changes in the immune response in vaccinated pigs. This is also supported by the immunization of pigs from group 3 with the recombinant plasmid pUBB76A-CD2v led to the formation of virus-specific T-lymphocytes.

Data on the immunogenic properties of CD2v is contradictory. EP402R gene deletion in the ASFV strain KK-262/C led to the full cancellation of protective properties in relation to the homologous virulent isolate Congo-49 (seroimmunotype II) [45]. Similarly, the deletion of CD2v and C-type lectin genes, or only the CD2v gene, considerably reduced the protective potential of ASFV-G-∆9GL [46].

In contrast, the removal of CD2v from the virulent Badajoz-71 isolate led to the appearance of a weakened virus, which provided protection not only against a homologous parental virus but also the heterologous isolates Spain-75 and Armenia-2010 [26]. The avirulent non-hemadsorbing virus ASF strains OURT88/3 and NHP68, presumably lacking CD2v/C-type lectin proteins, protected pigs from death after infection with the homologous ASF virulent virus [47,48].

A study by Borca and colleagues demonstrated that deletion of the 8DR gene from the genome of ASFV Georgia2010 isolate (ASFV-G-∆8DR) does not significantly alter the virulence of the virus. ASFV-G-∆8DR inoculated intramuscularly or intranasally produced clinical disease in domestic pigs indistinguishable from that induced by the same doses of the virulent parental ASFV Georgia2010 isolate. In addition, viremia values in ASFV-G-∆8DR do not differ from those detected in animals infected with the parental virus [49]. The authors are right that deleting a specific gene may have a different effect on the virus phenotype depending on the part of the genetic background where the deletion is performed. This is consistent with our hypothesis that the serotype-specific CD2v (gp 110-140) exposed on the membrane of infected macrophages may be dominant in the induction and implementation of cell-mediated CTL immune response for ASF protection. Deletion or defects in CD2v may reduce the value of CTL in forming protection against ASF. As a result, there is likely a redistribution towards an increased role of ADCC mechanisms and normal T lymphocytes in immunological protection against ASF. Therefore, non-haemadsorbing natural isolates or recombinants with CD2v deletion may exhibit the ability to form heterotypic protection [27,50].

## 5. Conclusions

Thus, our attempt to create balance protection against the Mozambique-78 strain (seroimmunotype III) by induction of both humoral factors of immunity (by vaccination with strain FK-32/135) and serotype-specific cellular immunity (by immunization with the plasmid pUBB76A_CD2v) was not successful. Nevertheless, our applied methodological approach can help study the reasons for forming homologous and heterologous immune protection at ASF.

## Figures and Tables

**Table 1:** Experimental groups of animals for assessment of immunogenicity and protective efficacy of vaccination by the ASFV strain FK-32/135 and/or pUBB76A_CD2v plasmid construct expressing CD2v protein from strain MK-200 and monoubiquitin B.

Group No.	Pig No.	Vaccination with FK-32/135 on Day 0	Immunization with pUBB76A_CD2v on Days 3 and 17	Infection with Virulent ***ASFV on Day 24	
				France-32 (Seroimmunotype IV)	Mozambique-78 (Seroimmunotype III)
1	1	*+	−	+	−
	2	+	−	+	−
	3	**−	−	+	−
	4	−	−	+	−
2	5	+	+	−	+
	6	+	+	−	+
	7	+	+	−	+
	8	+	−	−	+
	9	−	−	−	+
3	10	−	+	−	+
	11	−	+	−	+
	12	−	+	+	−

Note: *+ received injection; **− did not receive injection; ***ASFV–African Svine Fever Virus.

## Data Availability

The data presented in this study are available on request from the corresponding author.
